# Possible sexually dimorphic role of miRNA and other sncRNA in ASD brain

**DOI:** 10.1186/s13229-017-0117-0

**Published:** 2017-02-07

**Authors:** Cynthia M. Schumann, Frank R. Sharp, Bradley P. Ander, Boryana Stamova

**Affiliations:** 1 Department of Psychiatry and Behavioral Sciences, University of California at Davis, School of Medicine, 2805 50th Street, Sacramento, CA 95817 USA; 2 MIND Institute, University of California, 2805 50th Street, Sacramento, CA 95817 USA; 3 Department of Neurology, University of California at Davis, School of Medicine, 2805 50th Street, Sacramento, CA 95817 USA

**Keywords:** Autism, microRNA, small noncoding RNA, Superior Temporal Sulcus, Auditory cortex, Myelin, Oligodendrocytes, Sex, Sexual dimorphism, miR-181, miR-338, miR-219, miR-125, miR-448, Postmortem human brain

## Abstract

**Background:**

Autism spectrum disorder (ASD) is sexually dimorphic in brain structure, genetics, and behaviors. In studies of brain tissue, the age of the population is clearly a factor in interpreting study outcome, yet sex is rarely considered. To begin to address this issue, we extend our previously published microarray analyses to examine expression of small noncoding RNAs (sncRNAs), including microRNAs (miRNAs), in ASD and in the control temporal cortex in males and females. Predicted miRNA targets were identified as well as the pathways they overpopulate.

**Findings:**

After considering age, sexual dimorphism in ASD sncRNA expression persists in the temporal cortex and in the patterning that distinguishes regions. Among the sexually dimorphic miRNAs are miR-219 and miR-338, which promote oligodendrocyte differentiation, miR-125, implicated in neuronal differentiation, and miR-488, implicated in anxiety. Putative miRNA targets are significantly over-represented in immune and nervous system pathways in both sexes, consistent with previous mRNA studies. Even for common pathways, the specific target mRNAs are often sexually dimorphic. For example, both male and female target genes significantly populate the Axonal Guidance Signaling pathway, yet less than a third of the targets are common to both sexes.

**Conclusions:**

Our findings of sexual dimorphism in sncRNA levels underscore the importance of considering sex, in addition to age, when interpreting molecular findings on ASD brain.

**Electronic supplementary material:**

The online version of this article (doi:10.1186/s13229-017-0117-0) contains supplementary material, which is available to authorized users.

## Background

Autism spectrum disorder (ASD) is one of a number of neurodevelopmental disorders that display sexual dimorphism, occurring more frequently in males, which affect brain structure, gene expression, pathways, function, and ultimately behaviors that will require individualized treatments [1–3]. Extensive evidence demonstrates sex differences in ASD brain [4–7]. Sex chromosomes may play a role, with the Y chromosome being a possible risk factor for ASD and X chromosome perhaps having a protective effect [3]. Females appear to have a higher threshold for being affected by genetic factors than males, thus requiring a greater genetic burden, and may have greater brain plasticity [8, 9]. Females carry a higher proportion of de novo CNVs (copy number variants) than males, the CNVs in females disrupt a larger number of genes than in males, and females carry a greater number of de novo single nucleotide variants (SNVs) than males [10, 11]. Environmental and hormone factors that differ between the sexes, like testosterone, may impact the time course and severity of symptoms [12].

Few molecular studies of ASD brain tissue to date have considered potential sexual dimorphism often because of limited tissue availability of female cases. In typical brain development, male-biased gene expression changes are enriched for extracellular matrix, immune response, chromatin, and cell cytoskeleton pathways that have been implicated in ASD [13]. Sex differences in microRNA (miRNA) expression in the frontal cortex have also been described in typical neurodevelopment [14]. Specific genetic mechanisms, such as the expression of retinoic acid-related orphan receptor alpha (RORA) in the frontal cortex, which regulates CYFIP1, may be related to elevated testosterone levels and a potential contributor to the sex bias [15]. There is no clear evidence to date for systematic sex-differential expression of ASD risk genes in human brain; however, genes expressed at higher levels in males are significantly enriched for genes upregulated in postmortem autistic brain, including astrocyte and microglia markers [16].

Here, we extend our analyses of previously published microarray data [17, 18] to examine sexual dimorphism of microRNA and other small noncoding RNA (sncRNA) in male and female ASD and control brain tissue. We focused on two temporal cortical regions: the superior temporal sulcus (STS), a region implicated in social impairments in ASD [19–22], and the primary auditory cortex (PAC). Predicted miRNA targets were identified as well as pathways in which they over-populated. We then evaluated our findings in relation to the SFARI database and two gene expression studies in ASD brain: Ziats and Rennert, 2013 [13] and Werling et al., 2015 [16].

## Methods

### Brain samples

**Fig. 1:**
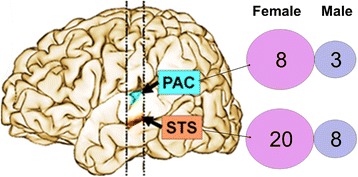
Dysregulated sncRNA in ASD females vs control females and ASD males vs control males for the STS (superior temporal sulcus) and the PAC (primary auditory cortex)

The methods used in the current study are similar to our recent publications [17, 18] (Table 1). Briefly, a total of 34 samples were obtained from 10 ASD and 8 control subjects. The PAC sample was taken from the crown of Heschl’s gyrus and included Brodmann’s areas, 41 and 42 (Fig. 1). The STS samples included Brodmann’s area 22 and were taken from the upper wall of the STS opposite Heschl’s gyrus (Fig. 1). Postmortem tissue integrity, processing, and microarrays are as described in our prior publications on this data set [17, 18].

**Table 1:** Subject characteristics for ASD (autism spectrum disorders) and controls

Primary diagnosis	Case number	Sex	Age (years)	Diagnostic measure	PMI (hours)	Primary cause of death
ASD	B-7002	F	5	ADI-R	33.0	Drowning
ASD	B-5342	F	11	ADI-R	12.9	Drowning
ASD	B-7575	M	15	Suspected ASD	30.8	Head trauma
ASD	B-6640	F	29	ADI-R	17.8	Seizure/stroke
ASD	B-7762	M	30	Suspected ASD	22.9	Epilepsy
ASD	B-5173	M	30	ADI-R	20.3	Gastrointestinal bleeding/seizure
ASD	B-6401^a^	M	39	ADI-R	14.0	Cardiac tamponade
ASD	B-7085	F	49	Suspected ASD	21.1	Cancer
ASD	B-7376^b^	F	52	ADI-R	39.2	Unknown
ASD	B-7886	M	50	ADI-R	22.7	Aspiration/seizure
ASD mean	*n* = 10		31.0 ± 5.3		23.5 ± 2.7	
CTRL	B-6736	F	4	–	17.0	Acute bronchopneumonia
CTRL	B-7387	M	17	–	30.8	Asphyxia/hanging
CTRL	B-7738	M	24	–	35.5	Unknown
CTRL	B-7369	M	36	–	26.0	Possible pulmonary embolism/MI
CTRL	B-7835	F	39	–	25.3	Asphyxia/pneumonia
CTRL	B-7333	M	40	–	25.3	Hepatic encephalopathy
CTRL	B-8018	M	54	–	19.9	Unknown
CTRL	B-8155	M	58	–	20.5	Unknown
CTRL mean	*n* = 8		34.0 ± 6.5		25.0 ± 2.1	

Demographics of each brain donor. The average ± the standard error of the mean is provided for ASD and CTRL. There were no significant differences in sex, age, or PMI between ASD and CTRL groups

*ASD* autism spectrum disorders, *CTRL* typically developing control, *PMI* postmortem interval

^a^STS excluded

^b^PAC excluded

### Statistics

We used a mixed regression model including diagnosis, sex, age, region, and subject, and group*sex and group*sex*region interactions. To obtain estimates of variance components for our mixed model, we used restricted maximum likelihood estimation (REML), which is suitable for unbalanced designs (W.A. Thompson, 1962). REML optimizes the parameter estimates for the effects in the model. We used repeated measures to account for multiple regions measured within the same subjects (STS and PAC), and we made the SubjectID a random effect. Considering that our sample size was unbalanced between males and females, some identified differences may be due to this limitation. Statistical significance was defined as *P* ≤ 0.005 and absolute fold change ≥1.2. To account for normal sexual dimorphism, we compared ASD female to control female and ASD male to control male and overlapped the results to identify sexually dimorphic sncRNA expression specifically in ASD.

### Pathway analysis and overlap with other studies

Ingenuity pathway analysis (IPA) was used to identify predicted miRNA targets of mature miRNA of major isoforms with sexual dimorphism and to identify over-represented pathways and biological functions. Targets of stem-loop precursor miRNAs (pre-miRNA) were not considered in this pathway analysis, since only mature miRNA can affect gene expression. A Fisher exact test with a Benjamini-Hochberg corrected *P* < 0.05 was used for determining significance of pathway enrichment. The sexually dimorphic predicted mRNA target genes and pathways were compared to three other data sets: (1) autism risk genes from SFARI using the hypergeometric probability function *phyper* in R with population size set to all protein-coding human genes of 20,687 [23], (2) Ziats and Rennert [13] based upon a total pool of 656 pathways and 90 bio-functions and disease categories in the IPA Knowledge Database, and (3) Werling et al. [16], an adult brain (102 mRNA/14,246), a replication adult brain data set (50 mRNA/14,869), and a prenatal brain (303 mRNA/9,840) (*P* < 0.005, FC > |1.2|).

## Results

A total of 17 STS and 17 PAC samples were analyzed from 10 ASD and 8 control subjects (Table 1). There were no significant differences in average ages or postmortem interval (PMI) between groups (Table 1). All data and analyses are available in supplementary materials or upon request.

### Sexual dimorphism of sncRNA in ASD brain

**Fig. 2:**
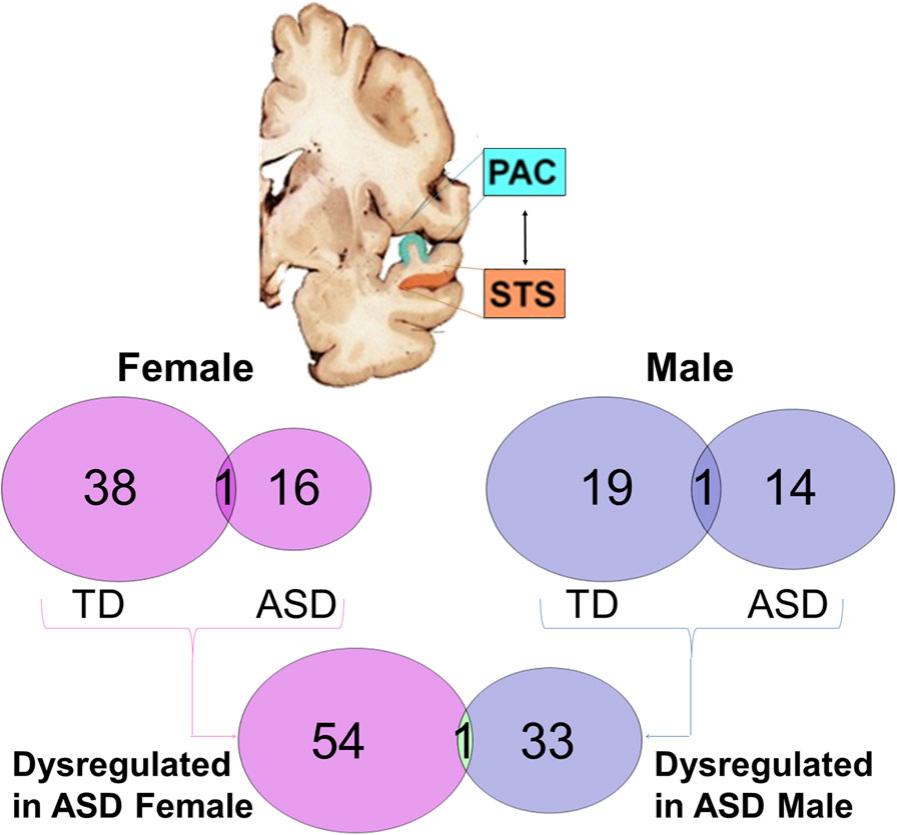
Regional sncRNA dysregulation in the superior temporal sulcus (STS) vs the primary auditory cortex (PAC) for female ASD and female control subjects and for male ASD and male control subjects

There are 20 sncRNAs differentially expressed (DE) in STS of ASD females compared to control females, which is significantly more (*P* = 0.04) than the 8 sncRNAs regulated in STS of ASD males compared to control males (Fig. 1, Table 2, Additional file 1: Table S1). There are eight sncRNAs DE in PAC of ASD females compared to control females, which was not significantly more than the three sncRNAs (*P* = 0.23) regulated in PAC of ASD male compared to control males (Fig. 1, Table 2, Additional file 1: Table S2). There are significantly more dysregulated sncRNAs in STS compared to PAC in ASD females, but not in ASD males (Fig. 1, Table 2). Regional analyses for STS vs PAC are shown in Fig. 2. There are 55 combined dysregulated sncRNAs in ASD females and 34 in ASD males (*P* = 0.03) (Fig. 2, Table 2, Additional file 1: Tables S3–S6). One overlapping sncRNA (mir-455) is DE in an opposite direction in ASD females and ASD males compared to control females and control males, respectively.

**Table 2:**
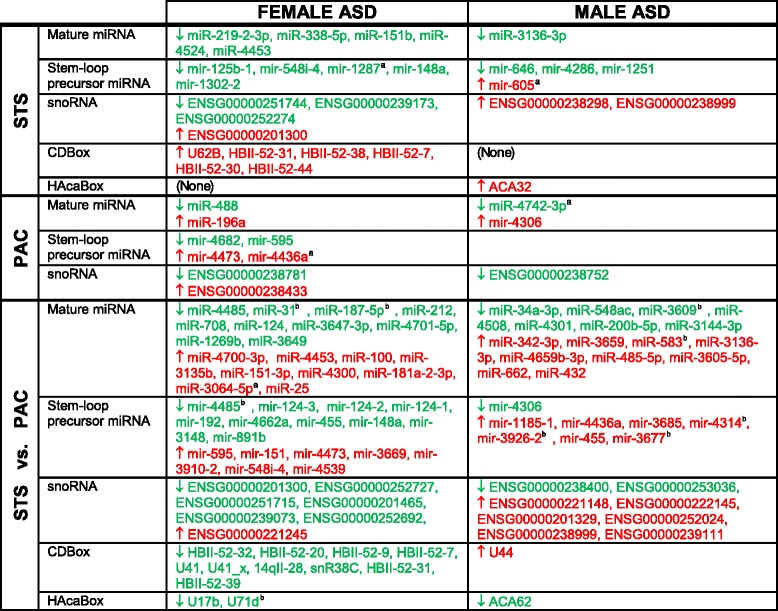
Dysregulated sncRNA in the temporal cortex of ASD male and female brains

^a^Noted in Ander et al., 2015

^b^Noted in Stamova et al., 2015

Green denotes downregulated sncRNA in ASD compared to control. Red denotes upregulated sncRNA in ASD compared to control

### Predicted mRNA targets and pathways

**Fig. 3:**
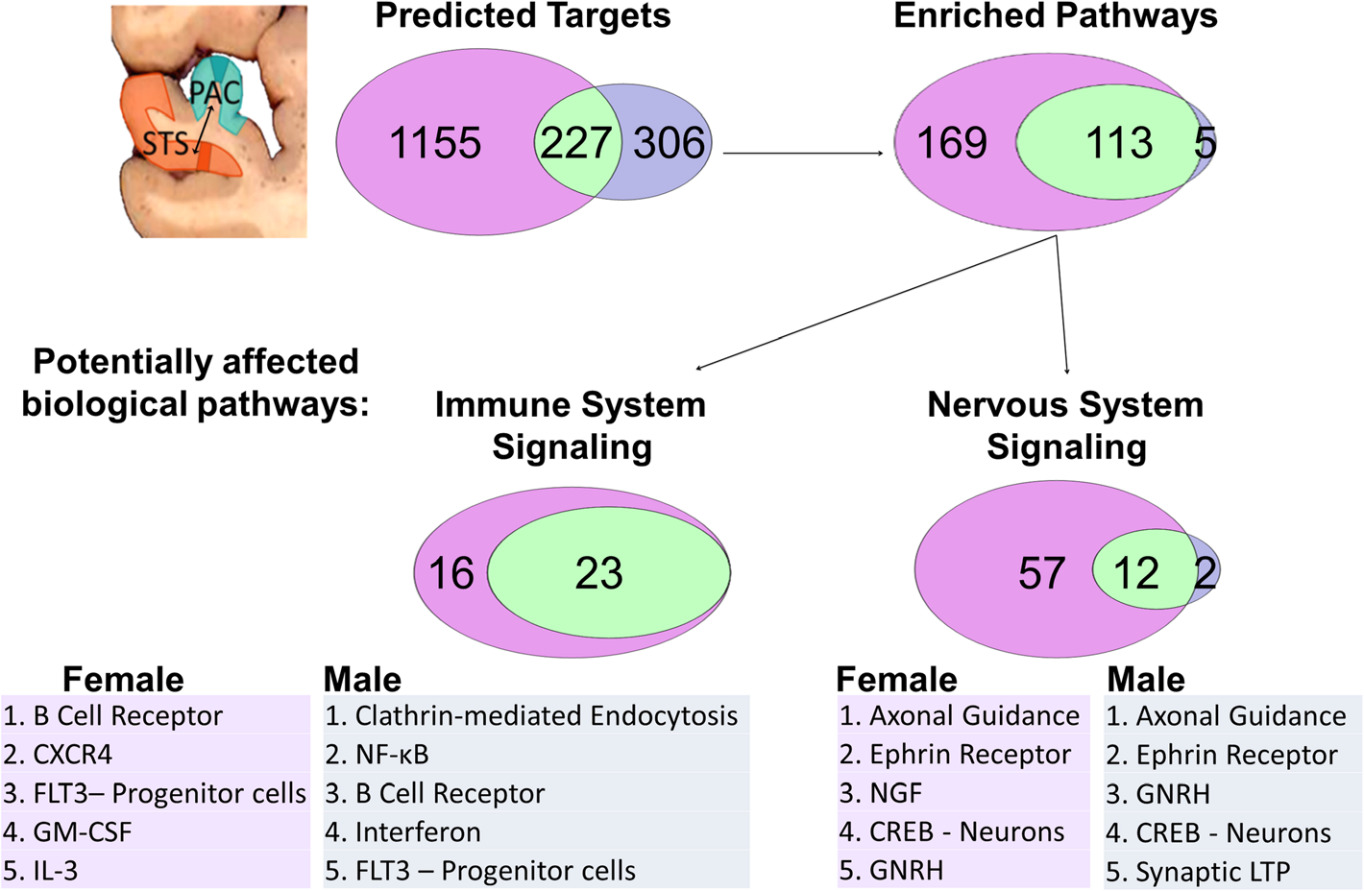
Predicted mRNA target genes and their enriched pathways for the sncRNA dysregulated in the regional STS to PAC comparison for female ASD and female control subjects combined (*pink*) compared to male ASD and male controls subjects combined (*blue*). The predicted targets and enriched pathways that are common to males and females are in *green*

**Fig. 4:**
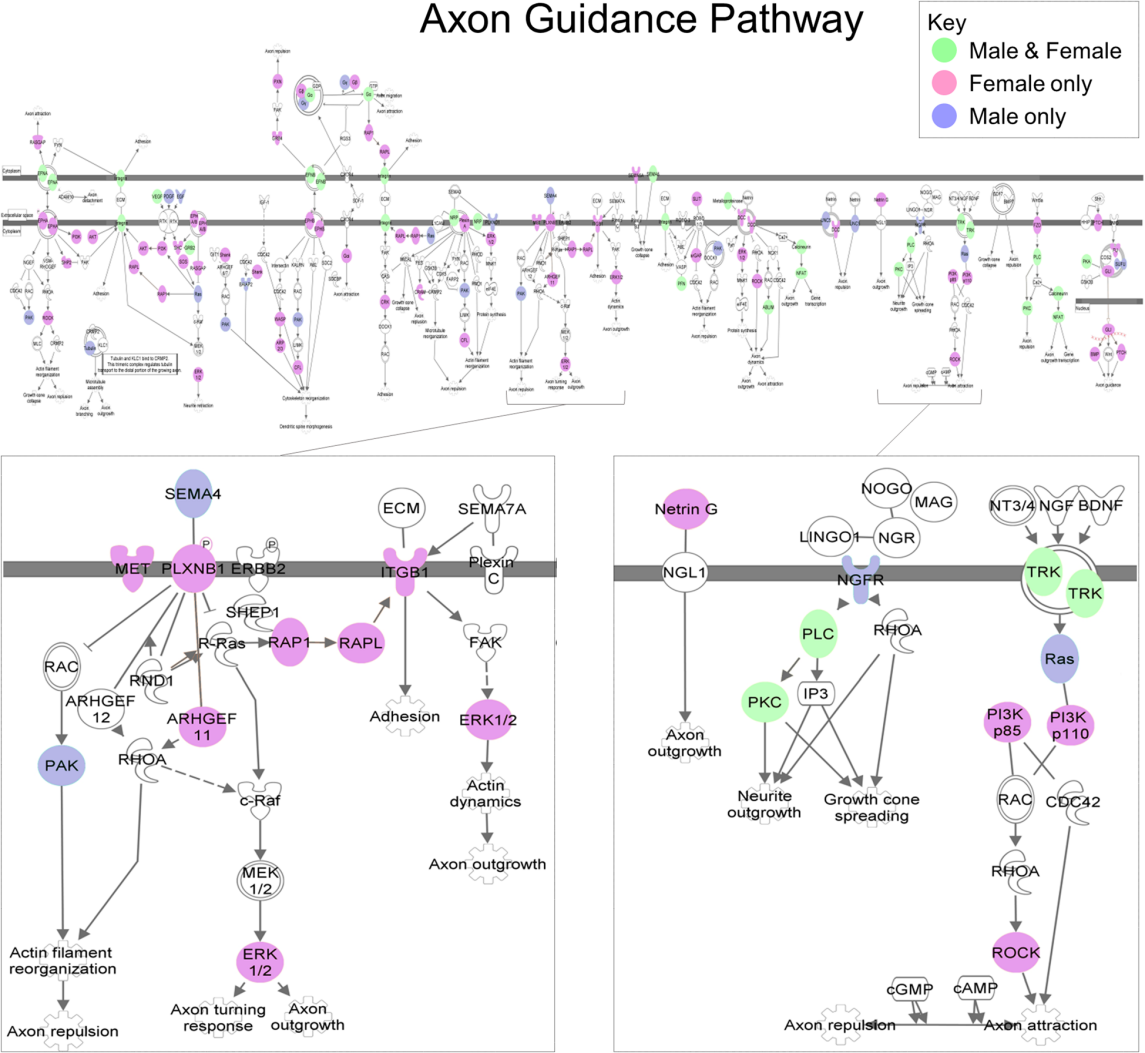
Sexual dimorphism in the axonal signaling pathway. Predicted targets of sexually dimorphic mature miRNAs that were dysregulated in regional analysis comparison of STS to PAC. *Pink* indicates targets predicted to be regionally dysregulated in ASD female only, *blue* are dysregulated in ASD males only, and *green* regulated in both males and females. Note that the majority of predicted targets are sexually dimorphic

Pathway analyses are summarized in Additional file 1: Table S7 and presented in Additional file 1: Tables S8–S17. There are significantly more over-represented pathways in ASD females than those in ASD males (*P* < 0.0001) in regional analysis (STS vs PAC), and the numbers of common pathways are also significant (*P* < 0.05) (Fig. 3). Of the total over-represented pathways in regional analyses, ASD females have significantly more immune, neurotransmitter, and other nervous system signaling pathways than those in ASD males (*P* < 0.0001) (Fig. 3). It is notable that even for common pathways, the specific target mRNAs are often sexually dimorphic. For example, both male and female target genes significantly populate the axonal guidance signaling pathway, yet less than a third of the targets are common to both sexes (Fig. 4).

### Overlap of the sexually dimorphic pathways with others implicated in ASD

We found a significant overlap between the predicted targets and pathways with the findings of Ziats and Rennert [13, 14] and SFARI risk genes (Additional file 2: Figure S1 and Additional file Tables 1: S18-S22). Seventy-four genes overlap between SFARI genes and our female regional analysis (STS vs PAC) and are significantly enriched in nervous system signaling, including glutamate receptor signaling, reelin signaling in neurons, and CREB signaling in neurons, as well as in p53 signaling. There is no significant overlap between our results and the three groups of Werling et al. [16]. However, we performed IPA pathway analysis on their 433 combined sexually dimorphic mRNA, and they are over-represented in 14 pathways. Of these, three overlap with our PAC pathways and nine overlap with our STS vs PAC pathways (trend towards significance, *P* < 0.1). In addition, miR-125 is sexually dimorphically dysregulated in the prefrontal cortex in Ziats and Rennert [14] and in the STS in our study (Table 2).

## Discussion

This short report underscores the importance of considering biological sex as a factor when interpreting gene expression studies of postmortem brains from individuals with ASD. Our findings from this small sample suggest that the expression of miRNA and other sncRNA is sexually dimorphic in the temporal cortex of ASD individuals. There are generally more dysregulated sncRNA, miRNA target genes, and pathways in ASD females compared to ASD males. There is a significant overlap between the male miRNA putatively dysregulated pathways in our study and that which occurs during normal male neural development [13]. Though there is an overlap between the female ASD regional STS-PAC miRNA targets and the SFARI genes, most of the sncRNA-regulated sexually dimorphic target genes are not enriched for autism risk genes, similar to the findings of Werling et al. [16]. The sexually dimorphic over-enrichment of miRNA target genes in the immune and nervous system pathways is consistent with prior gene expression studies of ASD brain [24, 25] that postulated that the immune pathways were related to environment and the neuronal pathways were related to genetics [24].

The greater number of dysregulated sncRNA, miRNA target genes, and pathways in females compared to male ASD subjects supports a body of evidence, suggesting that there is a greater genetic load in ASD females [9] [3, 26], and is consistent with a possible female protective effect [3, 27, 28]. The greater sncRNA dysregulation in females in our study might also support a recent proposal that female brains are less vulnerable to ASD because they are more plastic [8]. Our data could be interpreted to mean that the female ASD brain mounts a greater protective molecular response compared to males, factors that may contribute to the so-called “female camouflage effect” [29].

There are a number of sexually dimorphic miRNAs from our study that have been associated with ASD-relevant diseases and processes (Table 3). For example, one study of miRNA in serum of children with ASD [30] found two miRNAs, miR-151 and miR-181 that were also differentially expressed in the current study. miR-181 promotes synaptogenesis and decreases in axon growth [31, 32]. It is expressed in the brain and previously associated with autistic phenotypes [33] and schizophrenia [34]. The microRNA is also involved in an inflammatory response [35], influences apoptosis and mitochondrial function [36] in astrocytes, and targets GABA receptors [37].

**Table 3:** Relevant literature on example miRNAs with sexual dimorphism in ASD relative

miRNA	Relevant findings
miR-151 (female STS)	Regulated in serum of children with autism [30]
miR-181 (female STS vs PAC)	Regulated in serum of children with ASD [30]; expressed in brain, promotes synaptogenesis and decreases axon growth [31, 32]; associated with ASD phenotypes [33] and schizophrenia [34]; associated with inflammatory responses of astrocytes [35]; influences apoptosis and mitochondrial function in astrocytes [36]; targets GABA receptors [37].
miR-219 (female STS)	Regulates oligodendrocyte differentiation and likely myelin production [38]; regulates neural progenitors by dampening apical Par protein-dependent Hedgehog signaling [57]; polymorphisms in miR-219 affect genes involved in NMDAR signaling and schizophrenia [58]. Young age and environmental enrichment increase serum exosomes containing miR-219 that promote CNS myelination [59]; human endometrial-derived stromal stem cells (EnSCs) can be programmed into pre-oligodendrocyte cells by overexpression of miR-219 or miR-338 [60, 61].
miR-338 (female STS)	Regulates oligodendrocyte differentiation and likely myelin production [38]; attenuates cortical neuronal outgrowth by modulating expression of axon guidance genes and axonal mitochondrial genes [39–41]
miR-488 (female PAC)	Associated with panic disorder and regulate several anxiety candidate genes and related pathways [62]
miR-125 (female STS)	Differentially expressed in male vs female frontal lobe regions during normal neurodevelopment [14]; neuronal differentiation, and specifically promotes the generation of neurons of dopaminergic fate and possibly other types of neurons [63]

In STS of ASD female compared to control female, miR-219 and miR-338 showed the highest level of downregulation. Both miRNAs are involved in regulating oligodendrocyte differentiation and likely myelin production [38]. Though abnormalities of white matter have been observed in ASD brains for some time, recent studies point to important sex differences. For example, a recent anatomical study of autism brains showed large white matter regions showing significant sex × diagnosis interactions [5]. This was supported by sex differences found in the corpus callosum of young children with ASD [6]. While no alterations were observed in ASD males compared to control males, mean diffusivity, axial diffusivity, and radial diffusivity were all increased in ASD females compared to control females [6].

miR-219 and miR-338 both promote oligodendrocyte differentiation [38]. Inhibition of both miRNAs inhibits oligodendrocyte maturation and function in part by directly repressing negative regulators of oligodendrocyte differentiation, including transcription factors Sox6 and Hes5 [38]. miR-338 also attenuates cortical neuronal outgrowth by modulating expression of axon guidance genes and axonal mitochondrial genes [39–41]. However, it is the role of miR-219 and miR-338 on differentiation of oligodendrocyte precursors during development (with downregulation over fourfold in STS of ASD females, but not of ASD males) that may significantly contribute to the sexually dimorphic changes of white matter tracts including the corpus callosum seen in ASD brain [5, 6]. It is notable that decreased miR-219 and miR-338 were detected in female STS of ASD brains and not PAC, an important finding since STS is an association cortex implicated in social behavior, which is a core symptom of ASD, whereas PAC is not generally associated with ASD [42]. It will be important to examine miR-219 and miR-338 in amygdala and other brain regions implicated in ASD core symptoms, particularly since the number of oligodendrocytes in amygdala is decreased in ASD brain [43]. It will also be important to quantify oligodendrocyte numbers in STS of ASD brain given the current miRNA results.

It is important to note that sex differences in the miRNAome are prominent in health and disease [44–47]. Several miRNAs we identified in this study are also sexually dimorphic in other tissues and disease conditions. For example, miR-100, miR-196a, and miR-31 are sexually dimorphic in human amniotic mesenchymal stem cells (hA-MSCs) from obese versus normal weight women who gave birth to females, but not males [48]. This suggests that these miRNAs may be involved in metabolic changes. Additionally, miR-31 was sexually dimorphic in an animal model of systemic lupus erythematosus (SLE) [49]. Several mature miRNAs map to cytoband 14q32, where there is an imprinted miRNA cluster. In our study, miRNA-151b was downregulated in ASD-STS female vs typical STS female, while miR-342-3p, miR-432, and miR-485-5p were upregulated in ASD male in the inter-regional analysis compared to typical male. Loss of imprinting in this region has been associated with multiple diseases [50, 51]. Our results suggest involvement of miRNAs within or near this cluster in ASD as well.

As we have reported previously [17], there are changes of expression of sncRNA in ASD-PAC, though there are more changes in ASD-STS. In fact, some of the miRNAs we previously reported are included in the present study, indicating they may have been driven by sex differences as well. Interestingly, even though there were attenuated regional differences (STS vs PAC) in both male and female ASD compared to control (Fig. 2), the greatest number of miRNA targets by far was identified in the regional comparisons of STS to PAC in both females and males, though there were significantly more targets in females. These large differences of expression between regions, particularly between primary sensory cortices like PAC and association cortices like STS, have been noted previously in neurotypical adult and developing human brain [52, 53] and are consistent with the present data both for ASD and for controls. Integrative functional analyses of ASD risk genes implicate cortical layers II/III and V/VI pyramidal neurons [54], and co-expression network analyses of ASD risk genes also implicate layers V/VI cortical projection neurons [55]. The current findings of dysregulated miRNA that control oligodendrocyte differentiation in female STS, but not in male STS, adds complexity to the picture particularly since miR-219 and miR-338 are not dysregulated in either females or males in PAC. Thus, if projection neurons are involved in ASD, they are likely selectively affected in specific cortical regions and it is possible that there are regional differences of miRNA-mediated oligodendrocyte-myelination of the projection neuron axons that are also sexually dimorphic.

In general, there is little overlap of the *specific* miRNA regulated in ASD and control brains in this study and the miRNA described by Ziats and Rennert for typically developing human brain [14]. However, there is tremendous overlap of the predicted pathways that are sexually dimorphic. There is significant overlap for the pathways for males in our regional analyses and male pathways in neurotypical brains [13] and a trend for overlap between the sexually dimorphic pathways reported by Werling et al. [16] and our study.

There are important limitations to consider when interpreting this data that are common to studies of postmortem human brains, including small sample size and variation in cause of death, postmortem interval, age at death, agonal state, postmortem RNA integrity, and tissue preparation at different brain banks. In addition, the changes in miRNA and predicted targets and pathways studied here could lead to some aspects of ASD, but they could just as well be a consequence of the condition and even represent compensatory mechanisms. Future studies will need to assess both miRNA and mRNA in the same samples, so that one can determine if specific miRNA target mRNA are present and are regulated in an inverse direction as occurs for most miRNA-mRNA interactions. Small sample size is also limitation of this report and findings need to be confirmed in larger cohorts and with alternative gene expression profiling techniques such as qRT-PCR. However, this study clearly indicates that sex needs to be considered when interpreting data on postmortem human brains in ASD.

## Conclusions

Our own molecular studies, and many others, have demonstrated clear age-related changes in the brains of both typically developing and autistic individuals [17, 18, 24, 56]. Beyond age considerations, the current data also support a very important role for biological sex and suggest that pharmacological treatments would likely have to be evaluated separately in both sexes [3, 13, 15, 16]. A greater number of dysregulated sncRNAs and their gene targets and pathways in female ASD brain are consistent with a greater genetic load in females, a female protective effect, and possibly greater plasticity of female ASD brain. Although speculative, there are specific miRNAs dysregulated in STS of female ASD brain associated with oligodendrocyte differentiation (miR-219 and miR-338) that could relate to sexual dimorphism of white matter tracts, miR-488 that could relate to more anxiety in females, and miR-125 and miR-181 implicated in neuronal development that may be sexually dimorphic.

## Additional files

Additional file 1: Supplementary Data Tables. Table S1. sncRNA differentially expressed in ASD-STS vs CTRL-STS. Table S2. sncRNA differentially expressed in ASD-PAC vs CTRL-PAC. Table S3. Female (F) ASD Regional Analysis. F_ASD-STS vs F_ASD-PAC. Table S4. Female (F) controls (CTRL) regional analysis. F_CTRL-STS vs F_CTRL-PAC. Table S5. Male (M) ASD regional analysis. M_ASD-STS vs M_ASD-PAC. Table S6. Male (M) controls (CTRL) regional analysis. M_CTRL-STS vs M_CTRL-PAC. Table S7. Summary of putative target analyses. Table S8. Pathway analysis of putative targets of the mature miRNA from the PAC analysis in females. Table S9. Pathway analysis of putative targets of the mature miRNA from the PAC analysis in males. Table S10. Pathway analysis of putative targets of the mature miRNA from the STS analysis in females. Table S11. 227 common targets between female and male regional analyses. Table S12. Pathway analysis of putative targets of the mature miRNA from the female regional analysis. Table S13. Pathway analysis of putative targets of the mature miRNA from the male regional analysis. Table S14. Pathway analysis of putative targets of the mature miRNA from the female regional analysis: immune pathways. Table S15. Pathway analysis of putative targets of the mature miRNA from the male regional analysis: immune pathways. Table S16. Pathway analysis of putative targets of the mature miRNA from the female regional analysis: Neurotransmitter and other nervous system signaling pathways. Table S17. Pathway analysis of putative targets of the mature miRNA from the male regional analysis: neurotransmitter and other nervous system signaling pathways. Table S18. Pathway overlapping between our male regional analysis and the male-enriched pathways from Ziats and Rennert (Ref 15). Table S19. Seventy-four ASD-implicated genes amongst the 1382 putative targets of the miRNAs from the female regional analysis. Table S20. Canonical pathway enriched in the 74 ASD-implicated genes from the predicted targets in the female regional analysis Table S21. Targets overlapping between our analysis and the sexually dimorphic mRNA from Werling et al, Nature Communications, 2016; Supplementary Material: ncomms10717-s2. Table S22. Pathway overlapping between our male and female regional analysis and pathways were derived based on combined sexually dimorphic mRNAs from Werling et al, Nature Communications, 2016; Supplementary Material: ncomms10717-s2. (XLSX 130 kb)

Additional file 2: Supplementary Figure. Overlap with relevant studies. A Overlap between our male regional analysis (118 pathways) and Ziats and Rennert, Mol. Autism, 2013, male-enriched pathways (10 pathways); *P* of overlap = 0.0034. B Overlap between all our sexually dimorphic pathways (290 pathways) and Ziats and Rennert, Mol. Psychiatry, 2014, sexually dimorphic pathways (204 pathways); *P* of overlap < 0.00001. C Overlap between all our sexually dimorphic pathways (287 pathways) and Werling et al, Nat. Communications, 2016, sexually dimorphic pathways (14 pathways). *P* of overlap between Werling et al, 2016 and our regional analysis = 0.098, *P* of overlap between Werling et al, 2016, and our PAC analysis = 0.092. D Overlap between the predicted targets from our female regional analysis (STS vs PAC) (1382 targets) and SFARI ASD-implicated genes (768). *P* of overlap < 0.001. (TIF 48 kb)

## Abbreviations

ASD: Autism spectrum disorders
BA: Brodmann’s Area
CNV: Copy number variant
F: Female
M: Male
miRNA or miR-: microRNA
PAC: Primary auditory cortex of temporal lobe
PMI: Postmortem interval
RNAseq: RNA sequencing
sncRNA: Small noncoding RNA
SNV: Single nucleotide variant
STS: Superior temporal sulcus of temporal lobe
