# Supplementary figures and images for: Possible sexually dimorphic role of miRNA and other sncRNA in ASD brain
Source: Mol Autism. 2017 Feb 7;8:4. doi: 10.1186/s13229-017-0117-0 (PMC5294827; doi:10.1186/s13229-017-0117-0)

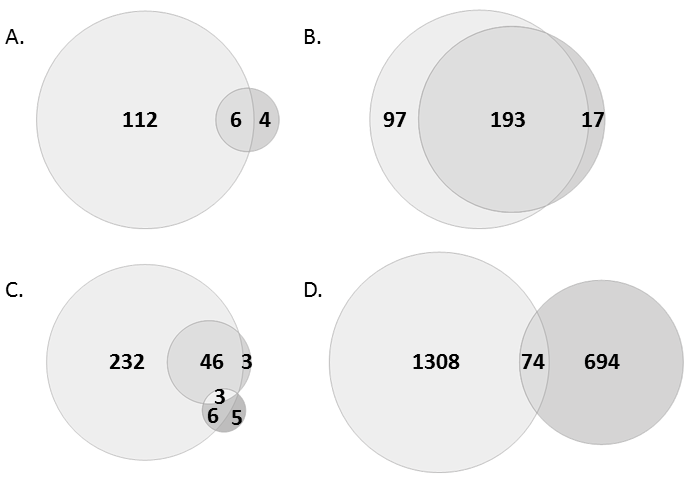

Supplement: Supplementary file 2 — Supplementary Figure. Overlap with relevant studies. A Overlap between our male regional analysis (118 pathways) and Ziats and Rennert, Mol. Autism, 2013, male-enriched pathways (10 pathways); P of overlap = 0.0034. B Overlap between all our sexually dimorphic pathways (290 pathways) and Ziats and Rennert, Mol. Psychiatry, 2014, sexually dimorphic pathways (204 pathways); P of overlap < 0.00001. C Overlap between all our sexually dimorphic pathways (287 pathways) and Werling et al, Nat. Communications, 2016, sexually dimorphic pathways (14 pathways). P of overlap between Werling et al, 2016 and our regional analysis = 0.098, P of overlap between Werling et al, 2016, and our PAC analysis = 0.092. D Overlap between the predicted targets from our female regional analysis (STS vs PAC) (1382 targets) and SFARI ASD-implicated genes (768). P of overlap < 0.001. (TIF 48 kb) [file 13229_2017_117_MOESM2_ESM.tif]
